# Somalia: A Nation at the Crossroads of Extreme Poverty, Conflict, and Neglected Tropical Diseases

**DOI:** 10.1371/journal.pntd.0004670

**Published:** 2016-09-29

**Authors:** Annum Jaffer, Peter J. Hotez

**Affiliations:** 1 School of Medicine, Baylor College of Medicine, Houston, Texas, United States of America; 2 Sabin Vaccine Institute and Texas Children’s Hospital Center for Vaccine Development, National School of Tropical Medicine at Baylor College of Medicine, Houston, Texas, United States of America; 3 Department of Biology, Baylor University, Waco, Texas, United States of America; 4 James A. Baker III Institute for Public Policy, Rice University, Houston, Texas, United States of America; Yale School of Public Health, UNITED STATES

The 2014–15 Ebola outbreak occurred in Guinea, Liberia, and Sierra Leone not from coincidence but because these three countries suffered from catastrophic breakdowns in health systems due to years of atrocities and civil wars, together with forced human migrations and deforestation [1]. A similar set of circumstances has now been identified in ISIS-occupied conflict zones in the Middle East and North Africa, suggesting that this region as well might be the next “shoe to fall” in terms of serious infectious diseases outbreaks [2,3]. The same may now also be true for the troubled nation of Somalia.

The Federal Republic of Somalia is roughly the geographic size of Texas [4] and located on the horn of Africa (Fig 1).

**Fig 1:**
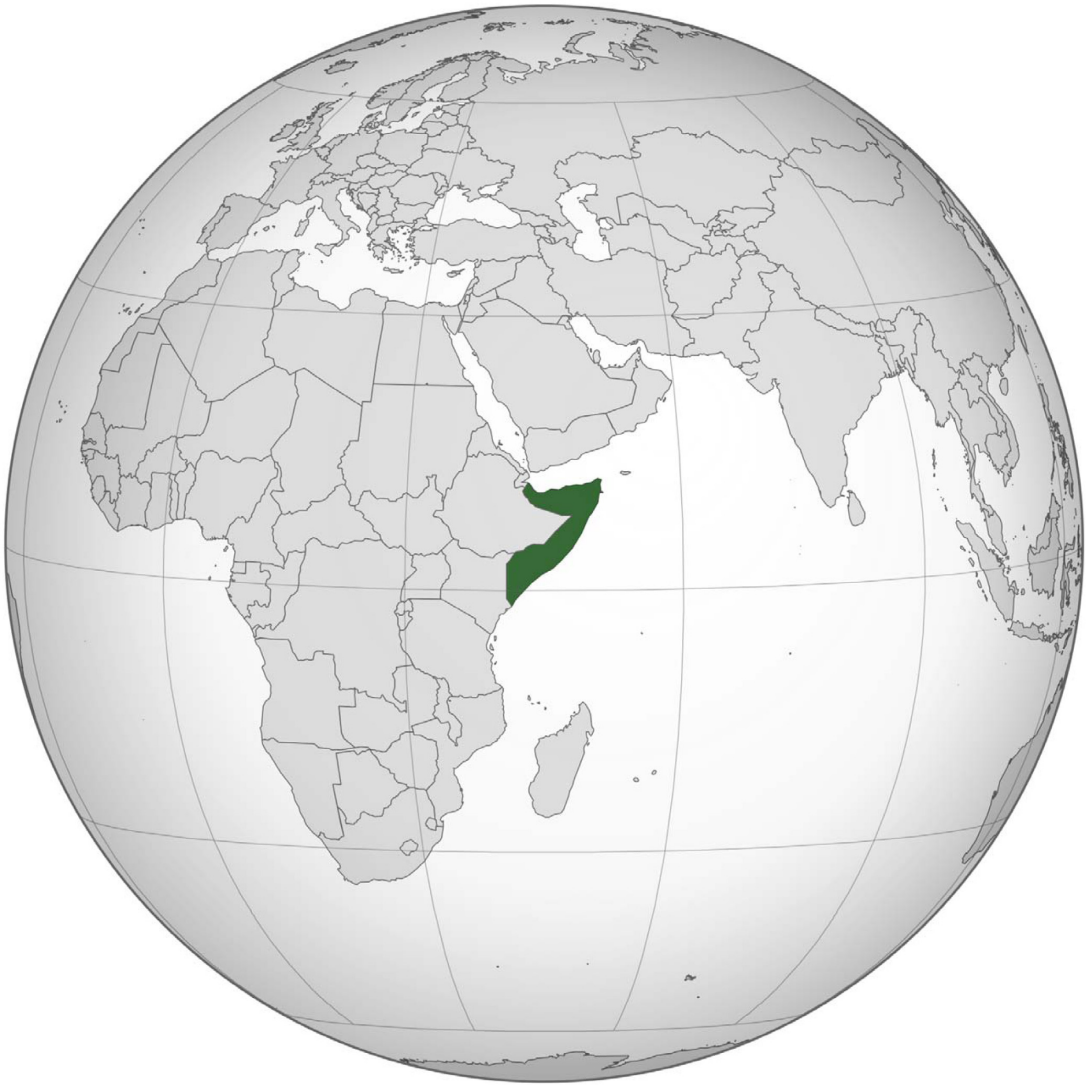
Federal Republic of Somalia. By Wikipedia contributor Flappiefh, available here: https://en.wikipedia.org/wiki/Somalia.

Today, the estimated 10.6 million people of Somalia live in one of the worst-performing economies in Africa, a situation that is exacerbated by frequent droughts and dust storms as a result of climate change [4]. According to the United Nations Development Programme (UNDP), more than 60% of Somalia’s population lives in severe poverty, and almost three-quarters of the population lives either in severe poverty or in near poverty [5]. One-third of Somalia’s children under the age of five are underweight [4]. Although a human development index (HDI) has not yet been calculated for Somalia because of an absence of quality data, there is every reason to believe that Somalia would be comparable to some of the worst-performing nations on the African continent.

For example, the life expectancy at birth in Somalia is 52 years [4], approximately the same as the three African countries listed at the bottom of the HDI scale—Chad, Central African Republic (CAR), and Democratic Republic of Congo (DRC). Similar to the situation in Somalia, civil warfare and instability have made it difficult for humanitarian aid to reach people in need in Chad, CAR, and DRC. For example, according to Médecins Sans Frontières (MSF) International, “DRC’s successive wars have had a severe impact on the health infrastructure and government-funded services.” [6] Humanitarian groups have been limited in providing health care and other basic needs to the people in CAR because of “looting of warehouses and aid convoys, threats to aid workers, and general insecurity…” [7]

## Neglected Tropical Diseases in Somalia

Based on World Health Organization (WHO) Parasitic Control and Transmission (PCT) databases, major NTDs (Table 1) flourish in this devastated setting [8–10]. Approximately three million school-aged children require regular and periodic mass treatment for intestinal worms, and 300,000 children require treatment for schistosomiasis [8,9]. Our calculated “worm index” of human development, a metric that inversely associates with HDI [11], is 0.318, which is lower than might be expected for a fragile nation state such as Somalia but can be partly explained by the absence of transmission of lymphatic filariasis, a key component of the worm index.

**Table 1:** The major NTDs in Somalia.

Population [4]	School-aged children requiring treatment for intestinal helminth infections in 2014 [8]	School-aged children requiring treatment for schistosomiasis in 2013 [9]	Population requiring treatment for lymphatic filariasis [10]	Total Worms	Worm Index^a^
10.6 million	3,079,697	293,206	None	3,372,903	0.318

^a^Calculated according to [11].

However, helminth infections are not the only important neglected disease in Somalia. Although minimal recent public health surveillance information is available, in terms of vector-borne diseases, it was estimated that there were between 1,400 and 2,700 cases of visceral leishmaniasis [12] and 114,617 “apparent” cases of dengue fever in 2010 [13]. Also, according to WHO, 70% of the population lives in areas classified as “high transmission” (>1 case per 1,000 population) for malaria, although only 10,470 malaria cases were confirmed in 2013 [14]. It is also known that tuberculosis is widespread [15,16], and Somalia is one of only seven African countries accounting for one-half of its recent cholera cases [17].

## A History of Conflict and Humanitarian Aid in Somalia

Somalia was established in 1960 following the joining of British and Italian Somaliland [4]. After the collapse of the regime of Mohamed Siad Barre in 1991, an outbreak of civil war left a decentralized and ineffective system of governance and a region in constant war. In the same year, Somaliland in the north declared independence from neighboring southern Somalia, creating a separate elected government, trained army and police force, capital city, and currency [18]. Shortly thereafter in 1998, Puntland, a region in the northeast, declared independence from Somalia, partly in order to separate itself from the civil unrest and warfare that were taking over the lives of its inhabitants [19]. Although both Somaliland and Puntland can claim a level of stability that southern Somalia cannot, according to Health Poverty Action, the many years of war before stability in the region has left it in a similar health care state as the rest of Somalia, ranking it amongst the worst in the world [20].

It is important to note that Somalia has been among the top ten recipients of humanitarian aid in seven of the last ten years ($905 million in 2012 alone) [21]. In the past, many aid organizations, including those affiliated with the UN, have been willing to provide health care access to the Somali people, and although care has been provided, it has not been as efficient and to the necessary extent. This has partly been because Somalia has seen many years of drought and violence, and different factions (including militant groups) have hindered the efforts of humanitarian aid organizations to properly vaccinate, deworm, and treat the Somali people.

Al-Shabab, founded in 2006, is a United States–designated Foreign Terrorist Organization that is fighting for the creation of its interpretation of a fundamentalist Islamic state. An example of a militant organization born in the time of civil war in Somalia, the group has largely contributed to warfare and unrest in the country [22].

Over the years, Al-Shabab grew in number, created links with other terrorist organizations, and gained control of most of southern and central Somalia [22]. When a famine plagued the nation between 2010 and 2012, the militant group exacerbated conditions by putting pressure on humanitarian aid organizations in controlling regions (the famine left 260,000 dead, half of which were under the age of 5) [23]. This included demanding up to $10,000 in taxes from each organization and attacking staff members [24].

In 2011 alone, Al-Shabab banned and/or raided 16 UN and international aid agencies, including the Office of the United Nations High Commissioner for Refugees (UNHCR), United Nations Children’s Fund (UNICEF), and WHO [25]. More than 2,000 violent incidents were reported by aid agencies in the first nine months of 2014 [26].

In 2013, MSF pulled out of Somalia after 22 years of providing access to health care services in the country (MSF vaccinated 58,620 people in Somalia in 2012 alone). This was necessitated by attacks on its staff and the subsequent death of a total of 16 staff members. At the time MSF pulled out of Somalia, more than 1,500 staff members were providing free primary health care, malnutrition treatment, epidemic response, immunization campaigns, and water (amongst other services) to the Somali people. According to Dr. Unni Karunakara, MSF’s international president in 2013, “these armed groups, and the civilian authorities that tolerate their actions, have sealed the fate of countless lives in Somalia” [27].

Reports show that in the “eight first months of 2015 alone, 85 security incidents involving humanitarians led to the death of 10, injury of 17, abduction of eight and arrest and detention of 34 aid workers” [28]. Recently, Al-Shabab has lost control of bigger towns and cities, but it still controls rural areas in southern and central Somalia [29].

Since the support of the African Union Mission in Somalia, government forces have been able to decrease Al-Shabab–controlled territory. Unfortunately, despite this gain for the people of Somalia, there are still “roadblocks and checkpoints in southern and central Somalia manned by armed actors” [28], which translates to a roadblock to aid. Adding to this, Al-Shabab continues to surround and restrict access to the territories it has lost in addition to its own controlled territory [28]. Still, there is hope for Somalia.

Despite all of the setbacks, assistance continues to be delivered to the Somali people by aid organizations. It is important to note that before MSF left in 2013, a high percentage of patients in some areas of Somalia had been successfully treated [16]. Moreover, while Somalia experienced an outbreak of polio in 2013–6 years after the last case was reported and 11 years after wild-type polio indigenous transmission was interrupted [30,31]—the nation demonstrated an ability to respond and prevent a recurrence, such that Africa recently celebrated its first year free of polio.

WHO is also committed to working in Somalia with five major strategic priorities, including expanding coverage for vaccine-preventable diseases; building capacity for reductions in HIV/AIDS, malaria, and tuberculosis; supporting the prevention and control of noncommunicable diseases; strengthening programs concerning reproductive, maternal, newborn, child and adolescent health; and health system strengthening, in addition to preparedness for outbreak and crisis responses [32]. However, so far there are no substantive or coordinated responses specifically for NTDs. The private End Neglected Disease (END) Fund has a track record of working in difficult and postconflict areas, including Cote d’Ivoire, Democratic Republic of Congo, Liberia, Mali, and Yemen [33], but there are currently no activities underway in Somalia.

## A Model for Optimism

Although many countries in Africa, including Somalia, are facing political instability, one specific country with a haunting history serves as a reason for hope—namely, Rwanda. After a sparked uprising in 1959, the country faced years of revolution and unrest that led to the displacement of almost 500,000 people by the end of the 1980s [34]. In the decades that followed, opposing militias within the country fought for power, and the 100-day genocide in 1994 led to the death of 20% of the population of the country [34,35]. In those few months, cholera spread amongst the people in Rwanda, 250,000 women were raped—leaving many HIV positive—and many humanitarian health care workers left while chronic infectious diseases from before the conflict continued to plague the nation, including malaria and tuberculosis [35,36]. Despite this bleak past, Rwanda has since doubled its life expectancy, while under-five child mortality, maternal mortality, and death rates for AIDS, tuberculosis, and malaria have decreased [35]. The Rwandan government achieved these gains by building a health care system based on disease-control programs, including training health care workers to treat diseases, promote family planning and immunizations, and refer patients to health centers where they can receive the care they need [36,37].

In light of Rwanda’s promising results, and with the comparison of the stability in current-day Somaliland, Puntland, and southern Somalia, it can be said that postconflict regions require more than political stability to turn around their massive health care setbacks. They need the leadership to build a system that can access, train, and manage health care workers. In most circumstances, this can only be done once barriers to the delivery of health care during civil war are lifted and there is a stable government that can implement organized goals and processes.

## Conclusion

In summary, NTDs and other tropical infections remain widespread in the extremely fragile nation-state Somalia. Until there are significant improvements in Somalia’s chronic complex emergency status, it is unlikely that this situation will experience substantial changes anytime soon. Learning from the active role the government of post-conflict Rwanda took to achieve remarkable gains in health, there is room for the leadership of Somalia and possibly Somaliland and Puntland to one day rebuild its health systems and infrastructure.
